# Interpersonal psychological needs as mediators linking abusive supervision and concurrent suicidal ideation: mediation analysis using parallel-process latent growth models based on a diary study

**DOI:** 10.1186/s40359-025-02966-9

**Published:** 2025-07-15

**Authors:** Yingying Yao, Zhihong Qiao, Dan Dong

**Affiliations:** 1 Counselling and Education Center, Student Affairs Department, Xiamen University, Xiamen, Fujian China; 2 Faculty of Psychology, Beijing Normal University, No. 19, Xinjiekouwai St, Haidian District, Beijing, 100875 China; 3 School of psychology, Fujian Normal University, Fuzhou, China

**Keywords:** Supervisor-student relationship, Abusive supervision, Suicidal ideation, Interpersonal psychological needs, Diary study

## Abstract

**Background:**

Given the frequent suicides among graduate students resulting from strained student-teacher relationships, we delved into the concurrent influence of abusive supervision on suicidal ideation among graduate students. Furthermore, we examined the potential mediating roles of the evolving trajectories of interpersonal psychological needs (thwarted belongingness and perceived burdensomeness) in this complex relationship.

**Methods:**

A diary study was conducted, involving 105 graduate students who had reported experiencing abusive supervision. Participants completed self-report surveys at twelve time points, assessing perceptions of daily abusive supervision, thwarted belongingness, perceived burdensomeness, as well as the presence of suicidal ideation. The study yielded 1,074 valid responses for analysis.

**Results:**

Employing a parallel-process latent growth model, we found that suicidal ideation exhibited parallel development with with abusive supervision, in both initial level (*β* = 0.714, *p <* 0.001) and growth rate (*β* = 0.618, *p <* 0.001). To investigate the underlying mechanisms, the latent growth curve mediation models were adopted to examine the potential mediating roles of interpersonal psychological needs. Specifically, initial levels of thwarted belongingness (*β* = 0.240, *p <* 0.001) and perceived burdensomeness (*β* = 0.362, *p <* 0.001) mediated the relationship between the initial levels of abusive supervision and suicidal ideation. However, only the growth rate of perceived burdensomeness showed marginal mediation (*β* = 0.027, *p* = 0.069) in the relationship between the growth rates abusive supervision and suicidal ideation.

**Conclusion:**

Daily abusive supervision significantly predicts concurrent suicidal ideation and this association is mediated by perceived burdensomeness. These findings highlight the importance of both fostering positive and supportive supervisory relationships, and addressing unmet interpersonal psychological needs to prevent and mitigate the adverse mental health consequences of abusive supervision.

## Introduction

### Background

Over the past decade, the mental health of graduate students, especially, the vulnerability to suicidal behaviour among them has garnered the attention of researchers [1]. Studies reveal alarmingly high rates of suicidal risk among graduate students, reaching 21.2% in United States [2] and 25.7% in China [1]. The crisis of suicide triggered by strained relationships between academic supervisors and graduate students has sparked widespread discussion and concern in recent years [3]. The persistent institutional hierarchies that reinforce power concentration — particularly through control of critical academic resources such as authorship, funding allocation, and research oversight —remain a primary driver of ongoing conflicts in supervisor-student relationships [4]. The concept of abusive supervision has been incorporated into educational setting to enhance our understanding of these relationships [5, 6].

Suicidal ideation experiences real-time fluctuations and substantial between-individual heterogeneity [7]. However, traditional cross-sectional studies [6] lack ecological validity in explaining these dynamic relationships and cannot account for individual differences. Emerging evidence indicates that nearly 40% of suicidal ideation variance occurs at the within-person level [8], suggesting that individuals’ suicidal risk states evolve meaningfully over short-term periods.

To better characterize the temporal dynamics of abusive supervision and suicidal ideation while controlling for between-individual differences, and to further elucidate their dynamic interaction over time, we employed a diary research design combined with parallel-process latent growth modeling to gain a deeper insight into their relationships.

### Abusive supervision and concurrent suicidal ideation

Abusive supervision originally conceptualized continuously verbal and non-verbal hostile behaviours perceived by a subordinate, including but not limited to bullying, intimidation, humiliation and sabotage, excluding physical contact [9, 10]. Persisting as a widespread social issue [11], abusive supervision, extends beyond formal employment relationship and has also been documented in other contexts, including supervisor-student relationships [6]. Persistent hostile behaviours exhibited by supervisors could trigger the suicidal risk. Within the framework of Conservation of Resources (COR) theory, abusive supervision produces two distinct harmful effects. Students might interpret the persistent hostility as an ongoing psychological threat, and prolonged exposure leads to chronic defensive states, progressively depleting emotional, cognitive, and self-evaluative resources. Abusive supervision triggers adverse proximal (short-term) and distal (long-term) consequences [12, 13]. Chronic abusive supervision results in psychological tension, and significantly increases the probability of emotional exhaustion [14], impairs mental health and welling-being of subordinates [15] and elevates suicidal risk [16]. Proximal variables like unmet psychological needs [17], depression [18], and loss of meaning in life [19] exacerbate these negative outcomes. Abusive supervision among college students worsens anxiety related to research projects [20], causes academic procrastination [21, 22], and leads to mental health challenges, including depression [5] and suicidal ideation [6].

The concurrent fluctuation of suicidal ideation has been demonstrated through ecological momentary assessment or diary studies [7, 23]. Similarly, abusive supervision fluctuates in real time, and daily exposure to abusive supervision affect concurrent emotional states, such as anger [24]. So, we employed a diary study and latent growth model to control the individuals’ differences. Hypothesis 1 posited that abusive supervision and suicidal ideation would demonstrate significant dynamic covariation. Specifically, both the initial status and growth rate of abusive supervision significantly and would positively predict the corresponding counterparts of suicidal ideation.

### Abusive supervision and thwarted belongingness

The empirically validated theory Interpersonal Psychological Theory of Suicide (IPTS) reinterprets suicide from the interpersonal perspective, emphasizing two proximal factors (thwarted belongingness and perceived burdensomeness) of suicidal ideation [25]. The unsatisfied states of interpersonal psychological needs associated with elevated suicidal ideation across the lifespan [26].

Thwarted belongingness denotes a psychologically painful mental state arising from unmet need for social belonging, characterized by loneliness and a absence of reciprocal care relationships, which can be aggravated by negative interpersonal interactions [27]. Authority figures’ interpersonal interactions significantly influence an employee’s psychological connectedness within the workplace, determining whether they experience a sense of belongingness within the group [28]. Abusive supervision, a destructive behavioural pattern, impairs the quality of leader-employee relationships, generating interpersonal frustration and systematically eroding belongingness, as confirmed by previous workplace studies. Abusive supervision weakens individuals’ attachment to the organization [29], induces the interpersonal withdrawal [30], reduces organizational citizenship behaviour [31] and prosocial behaviour among employees [32, 33].

Abusive supervision, an oppressive interaction pattern, hinders the fulfilment of related needs, and negatively correlates with the satisfaction of belonging needs [28]. Under an abusive supervision environment, subordinates may actively distance themselves from [14] or purposefully avoid contact with their superiors [34]. A study conducted in educational settings has also empirically validated the detrimental effects of abusive supervision on professional identity formation—a core dimension of belongingness [35].

Building on the adopted methodology, we proposed that the both the initial level and growth rate of abusive supervision would significantly and positively predict the corresponding initial level and growth rate of thwarted belongingness among graduate students (Hypothesis 2a).

### Abusive supervision and perceived burdensomeness

Perceived burdensomeness entails a subjective belief of incompetence or inefficiency, encompassing a sense of self-loathing or being a liability to other, subsequently elevating the suicidal risk [25]. Environmental factors are recognized as significant determinants either hinder or foster self-efficacy. Dysfunctional relationships within abusive environments undermine individual self-efficacy [36], as frequent criticism and mockery from supervisors makes ones feel that he/she are not up to the mark, exacerbating the sense of ineffectiveness. Previous studies have revealed that abusive supervision can reduce self-efficacy due to inadequate support and negative attitudes, leading to feelings of undervaluation [37–39]. Besides, abusive supervisors tend to overlook subordinates’ achievement and performance [40–42]. Under an abusive atmosphere, leader-employee exchange remains low, leading to maladaptive behaviours among subordinates, such as cyber-loafing [43] and surface acting, rather than deep acting [44].

Positive feedback plays a critical role in building self-efficacy by reinforcing competence beliefs and validating goal progress [36]. This constructive process enhances individuals’ perceived capability to execute tasks successfully, thereby promoting motivation and resilience. Conversely, abusive supervision, a destructive evaluative mechanism could diminish self-efficacy and increase the perceived burdensomeness by fostering feelings of being a liability to others.

Abusive supervision by academic supervisor, coupled with a persistent lack of academic guidance and resources causes a decline in graduate students’ self-efficacy and enhances perceived burdensomeness. So, we postulated that both the initial level and growth rate of abusive supervision would significantly and positively predict the corresponding initial level and growth rate of perceived burdensomeness among graduate students (Hypothesis 2b).

### Abusive supervision, interpersonal psychological needs and suicidal ideation

Drawing from IPTS, the interpersonal psychological needs (thwarted belongingness and perceived burdensomeness) function as critical proximal factors that mediate the relationship between distal stressors like abusive supervision and suicidal ideation [27]. Abusive supervision, acting as a prolonged stressor and threat for graduate students, has a significant impact on their mental health. The supervisor-student relationship is one of the pivotal interpersonal connections during their academic journey, and its quality deeply affects students’ belongingness. Perceived abusive supervision by students hinders their psychological needs satisfaction, estranging them from supervisors [10] and leads to avoidance of direct contact, worsening the relationship [34]. Furthermore, supervisors, as key evaluators of graduate students’ abilities, greatly influence graduate students’ self-perception of abilities, decrease self-efficacy, and exaggerate the perceived burdensomeness.

Empirical research confirmed abusive supervision depletes individuals’ resources, hinders the maintenance of interpersonal relationships [45], and compromises their ability to meet needs, leading to adverse outcomes including suicidal risk [26]. The strong link between interpersonal psychological needs and suicidal ideation has been well-established [25]. Meanwhile, a qualitative comparative analysis revealed that individuals belonging to the high-risk suicide group exhibited significantly higher level of interpersonal psychological needs [46]. A meta-analysis reinforced thwarted belongingness and perceived burdensomeness independently predicted suicidal ideation [26, 47]. A cross-sectional study examining the consequences of abusive supervision, suggested that interpersonal needs was parallel mediators in the effect of abusive supervision on suicidal ideation [6]. The convergence of qualitative, meta-analytic, and mediation analyses substantiates interpersonal needs as key mechanisms translating abusive supervision into heightened suicide vulnerability.

However, previous research is limited by methodological constraints, obscuring subject differences. To overcome these limitations, diary studies can be employed to track changes in abusive supervision, suicidal ideation, and interpersonal psychological needs over time. The study proposed that abusive supervision would affect the damage of interpersonal psychological needs and concurrent suicidal ideation, and the trajectories (represented by initial levels and growth rates) of thwarted belongingness (Hypothesis 3a) and perceived burdensomeness (Hypothesis 3b) serving as mediators between the trajectory of abusive supervision and that of suicidal ideation.

### Present study

This study examined the dynamic relationship between daily abusive supervision and concurrent suicidal ideation among graduate students, with a specific focus on the mediating roles of the trajectories of interpersonal psychological needs. Grounded in the Interpersonal Theory of Suicide (IPTS), we employed a diary study combined with parallel-process latent growth modeling to capture temporal dynamics and disentangle mediation mechanisms.

## Materials and methods

### Participants and sampling

We performed an online diary study targeting Chinese graduate students who could read Chinese, had weekly interactions with their supervisors, and reported experiencing abusive supervision in the entry assessment. This study focuses on a non-clinical sample of graduate students. Two groups will be excluded: (1) Individuals at high suicide risk, assessed by standardized protocols by clinical experts during the enrollment interview, as mandatory intervention protocols would compromise data validity; and (2) Participants reporting a schizophrenia diagnosis (single-item screening: “Have you ever been diagnosed with schizophrenia?”) for the potential symptom interference (e.g., hallucinations, cognitive deficits) might compromise questionnaire validity.

The final sample comprised 105 graduate students from 13 universities, with disciplinary distribution as follows: natural sciences (*n* = 59, 56.2%), humanities/social sciences (*n* = 45, 42.9%), and other disciplines (*n* = 1, 0.9%). The participants were 55.24% male, with a mean age of 23.42 ± 1.59, range from 20 to 28. Participant distribution across graduate years was: 39.1% first-year, 47.6% second-year, and the remainder third-year students.

### Procedure

The study comprised three phases: (a) recruitment and screening, (b) individual enrollment interviews with detailed explanations of research procedures and suicide risk protocols, and (c) a 28-day diary-study period. Participants received a base compensation of ¥100, with an additional ¥20 awarded for completing. more than 10 EMA prompt submissions. Approval for this study was granted by the Research Ethics Review Committee of Beijing Normal University.

#### Recruitment

Using snowball sampling and convenience sampling, we forwarded recruitment advertisements for the study in WeChat groups. Initially, 379 graduate students from 43 universities completed the screening survey. 265 of were excluded as they reported no prior experience with abusive supervision during their academic tenure.

#### Enrollment interviews

Qualified counselors (certified by the Chinese Psychological Society) performed structured enrollment interviews to: (1) evaluate participants’ recent suicide risk using standardized protocols, and (2) delineate risk management protocols. Exclusion criteria were met by one participant showing acute suicidality, with specific plan and strong suicidal impulses, triggering immediate crisis intervention. Three other candidates discontinued participation citing scheduling incompatibilities.

#### Diary-study period

Utilizing a WeChat mini-program, we delivered customized survey reminders aligned with participants’ academic calendars. Reminders were systematically dispatched three times per week over a 28-day period. Participants were instructed to complete the questionnaire three times per week over a 28-day period. with all responses required on the same day as prompt receipt. Five participants were excluded from the final analysis due to study withdrawal (providing fewer than three valid responses).

### Measures

#### Perceived abusive supervision

Four items were adapted from the original Abusive Supervision scale [9] and examples are as following: “the supervisor thinks I am an incompetent or ineffective student”, “the supervisor reminds me of my past mistakes and failures”. A 7-point Likert scale was used to measure the perceived intensity of abusive supervision since the last measurement, ranging from 1 (almost none or very weak) to 7 (very strong). Higher mean scores indicate the more severe of abusive supervision. The average internal consistency coefficient of the 12 measurements is 0.873, indicating good reliability.

#### Suicidal ideation

Four questions were selected to assess the suicidal ideation since the last measurement, examples are " I have thoughts of ending my life” and " I am actively planning to end my life”. Five-point scale (0-none, 1-very weak, 2-relatively weak, 3-strong, 4-very strong) was used, referencing previous studies [7]. Mean scores indicate suicidal ideation severity, with higher scores indicating stronger suicidal ideation. The average internal consistency coefficient of the 12 measurements is 0.858.

#### Interpersonal needs questionnaire

Six items were adapted from the Interpersonal Needs Questionnaire [27] and were rated using a 7-point Likert scale (1- completely incorrect, 7- completely correct), to assess thwarted belongingness and perceived burdensomeness since the last measurement. Example items included in the dimension of thwarted belongingness were “I feel like I belong (the reverse question) " and “I often feel like an outsider in social gatherings “, and example items for the dimension of perceived burdensomeness encompassed statements such as “the people in my life would be better off if I were gone " and “I think I am particularly useless”. Means were calculated for the two respective dimensions with higher scores indicating more serious deficiency in interpersonal needs. The average internal consistency of thwarted belongingness is 0.782, and of perceived burdensomeness is 0.851.

### Data analyses

**Fig. 1:**
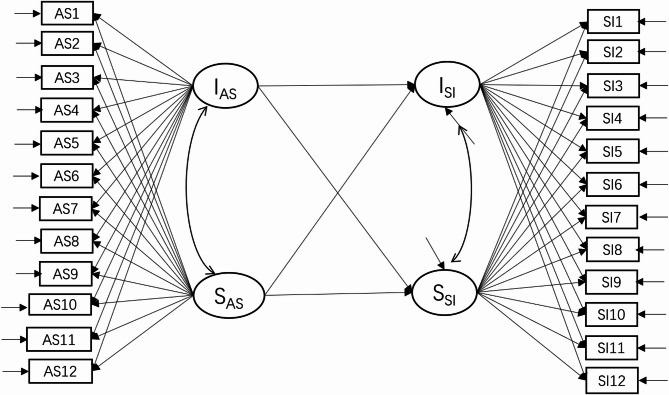
the parallel-process latent growth model of abusive supervision and suicidal ideation. Note: “I” means intercept factor and “S” means slope factor, the same below. AS = abusive supervision, SI = suicidal ideation

Firstly, the Unmeasured Latent Method Construct approach was employed used to test the common method biases and descriptive statistics, correlation was computed. Then, intraclass correlation coefficients (ICC) of these variables was examined. Secondly, we employed a parallel-process latent growth model (LGM) to examine the dynamic relationship between abusive supervision and suicidal ideation across 12 time points. The model estimated initial levels (intercept factor loadings = 1) and change rates (slope factor loadings = 0–11) for both variables (Fig. 1). Through path analysis, we specifically tested: (1) intercept-intercept correlations (baseline association), (2) slope-slope relationships (change-rate linkage), and (3) cross-construct paths (e.g., abusive supervision intercept → suicidal ideation slope), enabling comprehensive examination of their developmental trajectories and dynamic interrelationships.

**Fig. 2:**
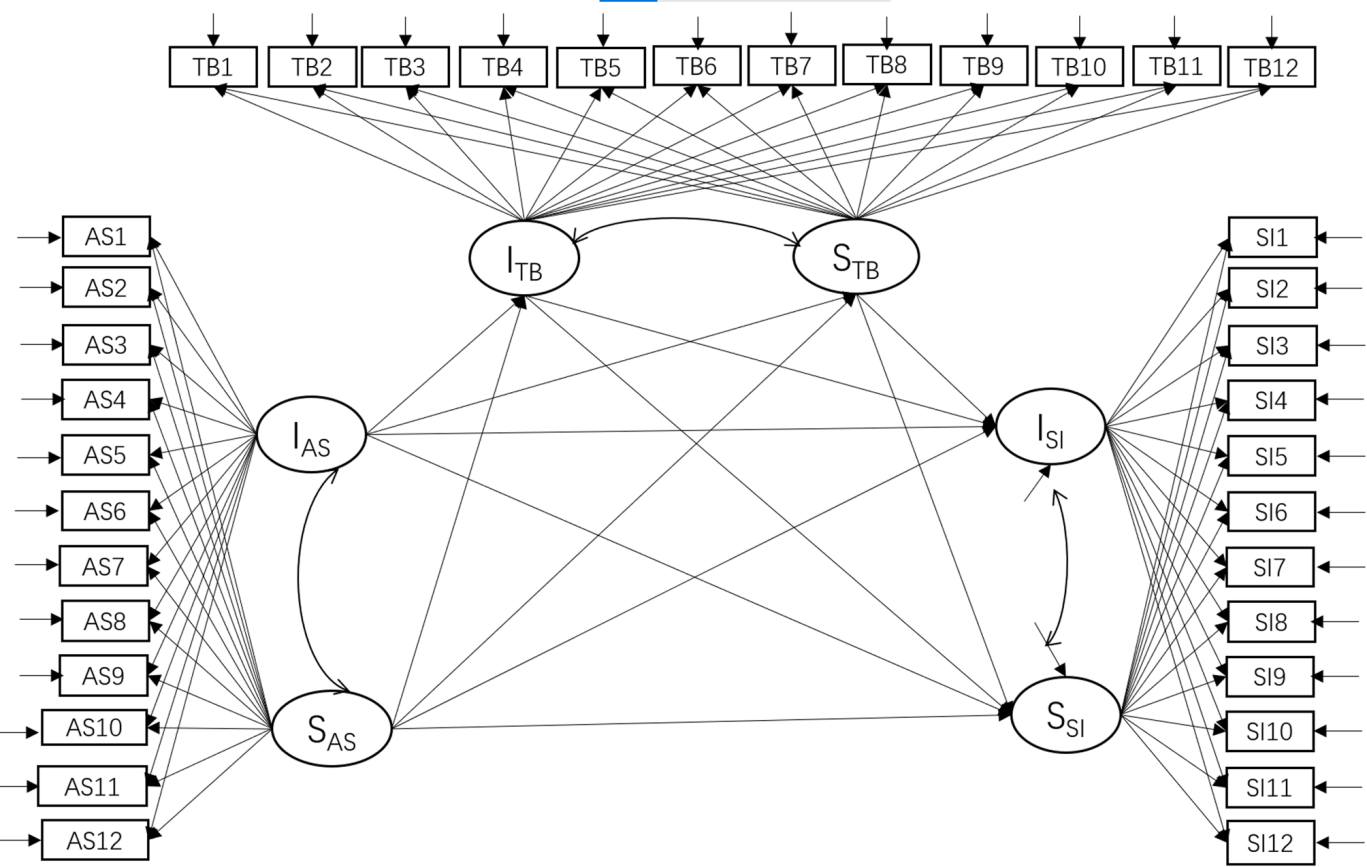
The latent growth curve mediation model with development trajectories of thwarted belongingness as mediation

Thirdly, building upon the model depicted in Fig. 1, the intercept and slope factors of thwarted belongingness or perceived burdensomeness were added as mediating latent variables. Latent growth curve mediation models were used to evaluate the mediation effects of interpersonal needs. Taking thwarted belongingness for example, the model is illustrated in Fig. 2.

Comparative Fit Index (*CFI*), Tucker-Lewis Index (*TLI*), Root Mean Square Error of Approximation (*RMSEA*), Standardized Root Mean Residual (*SRMR*) and the chi-square test were utilized to assess the fitness and adequacy of the models [48]. The aforementioned analyses were conducted utilizing SPSS 23.0 and Mplus 8.0.

## Results

### Descriptive and variability statistics

All questionnaires in our study were completed by independent subject reports, to assess potential common method biases, the Unmeasured Latent Method Construct (ULMC) approach was employed. Results from the confirmatory factor analysis indicated unacceptable model fit for the measured data, with *CFI* = 0.600, *TFI* = 0.547, *χ*^*2*^/*df* = 30.26, *RMSEA* = 0.196, *SRMR* = 0.133, suggesting no substantial common method bias in the measured data [49]. And the ICC of the all the variables were greater than 0.06, ranging from a minimum of 0.646 to a maximum of 0.837.

**Table 1:** Descriptive and variability statistics for variables

	Descriptive statistics				Variability statistics		
Variable	*M*	*SD*	*% nonzero*	*Skew*	*Intra-individual variation*	*Inter-individual variation*	*ICC*
Abusive supervision	3.240	1.529	93.5%	0.257	0.813	1.483	0.646
Thwarted belongingness	3.430	1.294	97.7%	0.398	0.372	1.226	0.767
Perceived burdensomeness	3.453	1.516	93.5%	0.194	0.543	1.695	0.757
Suicidal ideation	1.062	0.939	79.1%	0.562	0.139	0.712	0.837

### The co‑development of abusive supervision and suicidal ideation: the parallel process latent growth model

Parallel-process LGM of abusive supervision and suicidal ideation fits the data well (*χ*^*2*^/*df* = 2.045, *p* < 0.001, *CFI* = 0.906, *TLI* = 0.907, *RMSEA* = 0.100, *SRMR* = 0.075). the path coefficients for the model is presented in Table 2.

**Table 2:** Path coefficients for parallel-process LGM

		β	SE	*p*
variance	I_AS_	1.236^***^	0.212	0.000
	S_AS_	0.008^***^	0.002	0.000
correlation	I_AS_ * S_AS_	0.122	0.162	0.451
	I_SI_ * S_SI_	-0.059	0.217	0.785
pathways	I_AS_ → I_SI_	0.714^***^	0.068	0.000
	I_AS_ → S_SI_	-0.199	0.164	0.225
	S_AS_ → I_SI_	0.082	0.117	0.484
	S_AS_ → S_SI_	0.618^***^	0.172	0.000

**Note:** The uppercase letters I and S represent the intercept and the slope respectively. AS = abusive supervision, SI = suicidal ideation. ***p < 0.001, the same below

The intercept variance of abusive supervision was 1.236 (*p* < 0.001), demonstrating significant between-individual differences in initial levels. And the slope variance of is 0.008 (*p* < 0.001) confirming that growth rates differed significantly across individuals. However, no significant correlation was observed between these two variables (*r* = 0.122, *p* = 0.451). Similarly, no significant correlation was found between the intercept and slope of suicidal ideation (*r* = -0.059, *p* = 0.785). The initial level of abusive supervision and suicidal ideation did not affect the growth rate of them.

**Fig. 3:**
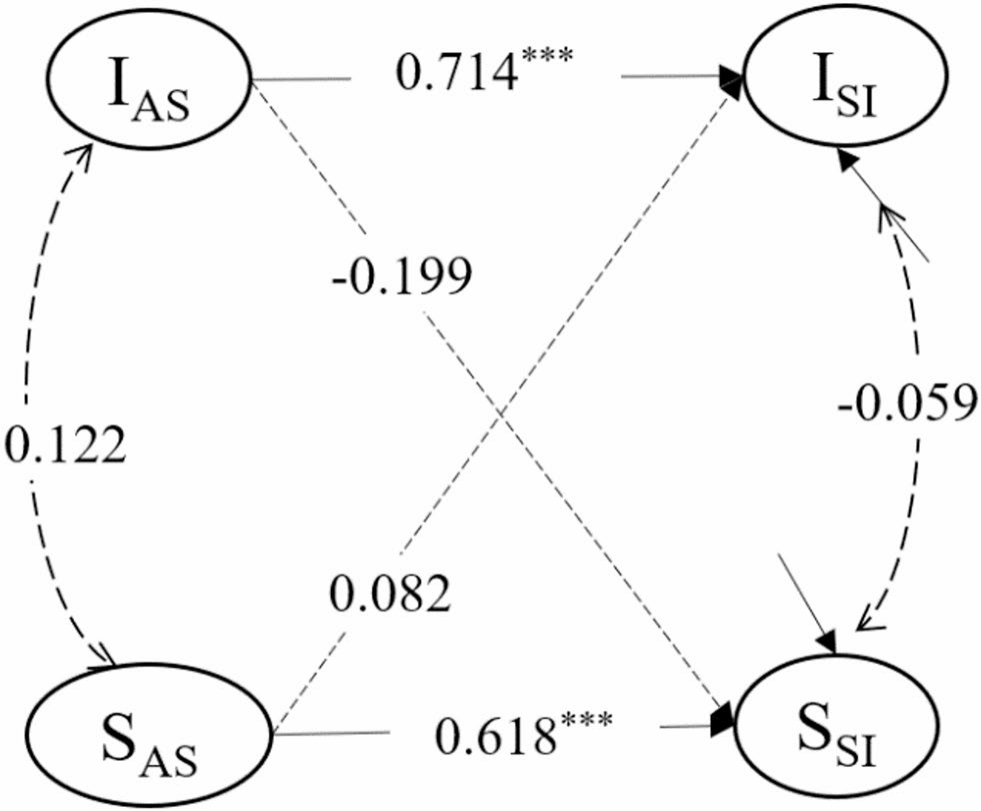
The parallel-process LGM of abusive supervision and suicidal ideation. **Note**: The graph showed only latent variable paths, with solid lines for significant relationships and dashed lines for non-significant ones. Same applies below

The intercept of abusive supervision served as a significant predictor for suicidal ideation (*β* = 0.714, *SE* = 0.068, *p* < 0.001), which means the higher initial level of abusive supervision, the higher initial level of suicidal ideation. The slope of abusive supervision predicted that of suicidal ideation (*β* = 0.618, *SE* = 0.172, *p* < 0.001), suggesting that individuals with steeper increases in abusive supervision also exhibited more pronounced growth in suicidal ideation over time. The abusive supervision and suicidal ideation exhibit a co-development relationship, both in terms of the initial level and the growth rate. The parallel-process LGM were shown in Fig. 3. Notably, supplementary analyses including gender and age as covariates yielded results nearly identical to our primary models (all effect size differences < 0.02, nonsignificant), demonstrating robust consistency in both direct and indirect effects. For simplicity, we report only the more streamlined models without control variables.

### The mediating effect of the trajectories of interpersonal needs: the latent growth curve mediation models

**Fig. 4:**
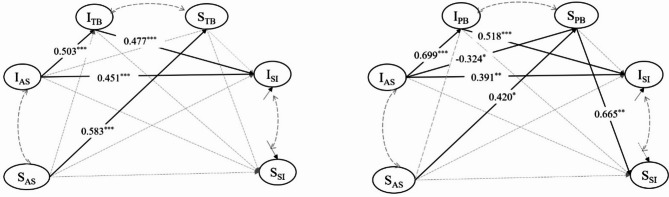
Path analysis of latent growth curve mediation models

Taking the intercept and slope of abusive supervision as predictors, and the intercept and slope of suicidal ideation as outcomes, latent growth curve mediation models mentioned above were tested. The latent growth curve mediation models fitted the data well (thwarted belongingness as mediator: *CFI* = 0.876, *TLI* = 0.877, *χ*^*2*^*/df* = 1.956, *p* < 0.001, *RMSEA* = 0.095, *SRMR* = 0.069; perceived burdensomeness as mediator: *CFI* = 0.868, *TLI* = 0.869, *χ*^*2*^*/df* = 2.004, *p* < 0.001, *RMSEA* = 0.098, *SRMR* = 0.070). Detailed pathways coefficients are presented in Table 3; Fig. 4.

**Table 3:** Path analysis of latent growth curve mediation models

		TB as Mediator			PB as Mediator		
		*β*	*SE*	*p*	*β*	*SE*	*p*
variance	I_AS_	1.230^***^	0.210	0.000	1.228^***^	0.211	0.000
	S_AS_	0.008^***^	0.002	0.000	0.008^***^	0.002	0.000
correlation	I_AS_ * S_AS_	0.146	0.160	0.359	0.139	0.164	0.397
	I_SI_ * S_SI_	-0.065	0.234	0.779	-0.128	0.303	0.674
pathways	I_AS_ → I_SI_	0.451^***^	0.099	0.000	0.391^**^	0.130	0.003
AS→SI	I_AS_ → S_SI_	-0.125	0.216	0.561	0.100	0.270	0.712
	S_AS_ → I_SI_	0.212	0.144	0.141	-0.009	0.122	0.939
	S_AS_ → S_SI_	0.327	0.294	0.266	0.347	0.231	0.134
pathways	I_AS_ → I_Mediator_	0.503^***^	0.093	0.000	0.699^***^	0.067	0.000
AS→Mediator	I_AS_ → S_Mediator_	-0.096	0.137	0.483	-0.324^*^	0.146	0.026
	S_AS_ → I_Mediator_	-0.117	0.126	0.351	0.068	0.119	0.569
	S_AS_ → S_Mediator_	0.583^***^	0.140	0.000	0.420^*^	0.176	0.017
pathways	I_Mediator_ → I_SI_	0.477^***^	0.080	0.000	0.518^***^	0.100	0.000
Mediator →SI	I_Mediator_ → S_SI_	-0.026	0.184	0.886	-0.129	0.215	0.548
	S_Mediator_ → I_SI_	-0.092	0.123	0.456	0.110	0.124	0.375
	S_Mediator_ → S_SI_	0.415	0.266	0.118	0.665^**^	0.230	0.004

Note: TB = thwarted belongingness, PB = perceived burdensomeness, ^***^p < 0.001, ^**^p < 0.01, ^*^p < 0.05, ^†^*p* < 0.1, the same below

#### The mediating effect of the trajectory of thwarted belongingness

We examine the mediating effect of the trajectory of thwarted belongingness. As presented in the Table 3, the intercept of abusive supervision significantly predicted the intercept of thwarted belongingness *(β* = 0.503, *SE* = 0.093, *p* < 0.001) and suicidal ideation (*β* = 0.451, *SE* = 0.099, *p* < 0.001). The slope of abusive supervision significantly is association with that of thwarted belongingness (*β* = 0.583, *SE* = 0.140, *p* < 0.001), but not the slope of suicidal ideation (*β* = 0.327, *SE* = 0.294, *p* = 0.266). Among the pathways from mediator to suicidal ideation, only the intercept of thwarted belongingness significantly and positively predicts that of suicide ideation (*β* = 0.477, *SE* = 0.080, *p <* 0.001).

The mediation effects were shown in Table 4, revealing a significant direct effect (*β* = 0.451, *SE* = 0.099, *p* < 0.001) of the intercept of abusive supervision (I_AS_) on that of suicidal ideation (I_SI_), as well as a significant indirect effect through the intercept of thwarted belongingness (*β* = 0.240, *SE* = 0.058, *p <* 0.001). When examining the mediating effect from the slope of abusive supervision (S_AS_) to the slope of suicidal ideation (S_SI_), neither the direct effect (*β* = 0.327, *SE* = 0.294, *p* = 0.266) nor indirect effect (*β* = 0.245, *SE* = 0.174, *p* = 0.158) were statistically significant. It was suggested that the initial level of thwarted belongingness partially mediated the relationship between the initial level of abusive supervision and that of suicidal ideation.

**Table 4:** The mediating effect test of the trajectories of interpersonal needs

		TB as mediator			PB as mediator		
		*β*	*SE*	*p*	*β*	*SE*	*p*
Pathways I_AS_ → I_SI_	direct effect						
	I_AS_→I_SI_	0.451^***^	0.099	0.000	0.391^**^	0.130	0.003
	indirect effect						
	I_AS_ → I_Mediator_ → I_SI_	0.240^***^	0.058	0.000	0.362^***^	0.074	0.000
	I_AS_ → S_Mediator_ → I_SI_	0.009	0.017	0.609	-0.036	0.046	0.437
	Total indirect effect	0.248^***^	0.064	0.000	0.327^***^	0.095	0.001
	Total effect	0.700^***^	0.071	0.000	0.718^***^	0.069	0.000
Pathways S_AS_→S_SI_	direct effect						
	S_AS_ → S_SI_	0.327	0.294	0.266	0.347	0.231	0.134
	indirect effect						
	S_AS_ → I_Mediator_ → S_SI_	0.003	0.021	0.885	-0.009	0.023	0.706
	S_AS_ → S_Mediator_ → S_SI_	0.242	0.164	0.140	0.027^†^	0.153	0.069
	Total indirect effect	0.245	0.174	0.158	0.270^†^	0.156	0.083
	Total effect	0.572^***^	0.185	0.002	0.617^***^	0.179	0.001

#### The mediating effect of the trajectory of perceived burdensomeness: the latent growth curve mediation model

We examined the mediating role of the trajectory of perceived burdensomeness. As evident in Table 3, the intercept of abusive supervision served as a significant predictor for that of perceived burdensomeness (*β* = 0.699, *SE* = 0.067, *p* < 0.001) and suicidal ideation (*β* = 0.391, *SE* = 0.130, *p* = 0.003). The slope of abusive supervision significantly foresaw that of perceived burdensomeness (*β* = 0.420, *SE* = 0.176, *p* = 0.017), but not that of suicidal ideation (*β* = 0.347, *SE* = 0.231, *p* = 0.134). Of the pathways leading to suicidal ideation, the intercept and slope of perceived burdensomeness strongly predicted those of suicidal ideation, with significant *β* values of 0.518 (*SE* = 0.100, *p* < 0.001) and 0.665 (*SE* = 0.230, *p* = 0.004), respectively.

As shown in the Table 4, the direct effect from the intercept of abusive supervision (I_AS_) to the intercept of suicidal ideation (I_SI_) was significant (*β* = 0.391, *SE* = 0.130, *p =* 0.003). Furthermore, the indirect effect mediated by the intercept of perceived burdensomeness (I_PB_) was also statistically significant (*β* = 0.362, *SE* = 0.074, *p <* 0.001). The direct effect from the slope of abusive supervision (S_AS_) to that of suicidal ideation (S_SI_) was not significant (*β* = 0.347, *SE* = 0.231, *p =* 0.134). However, the indirect effect mediated by the slope of perceived burdensomeness was marginally significant (*β* = 0.027, *SE* = 0.153, *p* = 0.069).

Combined with path analysis and mediating effects, the intercept of perceived burdensomeness partially mediated the effect of the intercept of abusive supervision on that of suicidal ideation, while the slope of perceived burdensomeness completely mediated the effect of the slope of abusive supervision on that of suicidal ideation.

## Discussion

### Discussion based on findings

Firstly, the study validated the co-development relationship between abusive supervision and suicidal ideation. Specifically, the initial level of abusive supervision perceived by graduate students was associated with the initial level of suicidal ideation, while the growth rate of abusive supervision was associated with the growth rate of latter. Hypothesis 1 thus supported. The current findings provides further evidence that abusive supervision, as a detrimental stressor, are significantly compromises individual mental health and may lead to suicidal ideation in extreme cases. Previous research has shown that the power asymmetry inherent in long-term abusive supervision can induce lasting alterations in the hypothalamic-pituitary-adrenal (HPA) axis, activating subsequent stress-related biological processes such as fear and tension, ultimately amplifying suicidal impulsivity [50]. Notably, cumulative exposure to abusive supervision can instantly induce worry and fear among graduate students. Repeated interpersonal failures and power imbalances in the supervisor-student relationship exacerbate these fears, may precipitate the onset of suicidal ideation.

Secondly, the study reinforced the validity of IPTS from a dynamic perspective, partially confirming the critical mediating roles of interpersonal psychological needs on a more granular temporal scale. Furthermore, within supervisor-student dyads, daily abusive supervision significantly increased perceived burdensomeness, displaying concurrent change with suicidal ideation. In the latent growth curve mediation models, the initial level of interpersonal needs (thwarted belongingness and perceived burdensomeness) functioned as mediators in the relationship between the initial level of abusive supervision and that of suicidal ideation. This finding confirms the pivotal role of interpersonal needs deficiency in suicidal ideation [6].

Regarding growth rates, only the perceived burdensomeness growth rate fully mediates the influence of abusive supervision on suicidal ideation. More rapid changes in abusive supervision associated with faster shifts in perceived burdensomeness, which in turn accelerate the progression of suicidal ideation. Research hypothesis 3b was confirmed. In the current study, we operationalize abusive supervision as graduate students’ perceived experience of sustained hostile treatment from their advisors, which primarily includes public demeaning, deliberate neglect, unwarranted blame, and resource deprivation [9, 51]. Students’ perceptions of abusive supervision exhibited by supervisors could lead to self-doubt during the critical stage of professional identity formation and development. Such supervisory behaviour could diminish graduate students’ professional efficacy [52] and increase suicidal risk via a chain mediation effect involving autonomy needs and professional identity [35]. As the primary evaluators of graduate students’ academic performance, supervisor who engage in continuous abusive supervision cultivates a perception among students that their abilities and achievements go unrecognized, and it could immediately and cumulatively erode students’ self-efficacy. The previous study found that others’ academic expectations positively impacted one’s suicidal ideation through a mediator of perceived burdensomeness [53]. Therefore, abusive supervision may undermine students’ self-efficacy, leading to increased perceived burdensomeness and ultimately elevating the risk of suicide.

However, this study failed to detect the mediating effect of the growth rate of thwarted belongingness. Two primary factors may explain this unexpected finding, the temporal insufficiency and potential peer buffering effects. Our 4-week measurement period may have been insufficient to adequately capture the chronic development of thwarted belongingness. Grounded in the Interpersonal Theory of Suicide, thwarted belongingness emerges through prolonged erosion of social connections; thus, our relatively short observation window may not have allowed thwarted belongingness’s deterioration to reach levels necessary for detectable mediation effects. In addition, protective dynamics within peer networks may have attenuated thwarted belongingness declines. When graduate students collectively endure abusive supervision, their shared negative experiences can strengthen in-group cohesion. These emergent support systems may temporarily preserve belongingness perceptions, creating a buffer that slows observable declines in belongingness.

The study makes some innovations and contributions. Firstly, it extends the concept of “abusive supervision” from workplace to academia, advancing our understanding of supervisor-student relationships. Secondly, we expanded the empirical research on IPTS through expanded sampling and a more ecologically valid methodology. Utilizing the diary study, this is the initial study to observe the concurrent influence of daily abusive supervision on suicidal ideation among non-clinical participants and to explore the mediating roles of the trajectories of interpersonal needs. And it greatly enhanced the persuasiveness and applicability of the IPTS. Thirdly, the research reveals the deleterious influence abusive supervision on suicidal ideation among graduate students, which not only ruins student-supervisor relationship but also triggers significant mental health issues. The research findings offer insights into how to prevent suicidal risk among graduate students. Supervisors should fundamentally reconceptualize their role —transitioning from an employment-based paradigm to collaborative academic partnerships— avoiding abusive behaviours and fostering equitable environments that support both scholarly development and psychological wellbeing, thereby serving as crucial interpersonal anchors throughout graduate training. Furthermore, the mediating role of interpersonal psychological needs prompts that mental health education measures focus on enhancing belongingness and reducing perceived burdensomeness may help to prevent the occurrence and development of suicidal ideation. For example, affirming graduate students’ competencies could effectively counterbalance the negative impact of abusive supervision. The proximal influence of interpersonal psychological needs underscores that the need to include their assessment in mental health screenings for graduate students and the deficiencies in these needs offers valuable insights for suicidal risk identification.

### Limitations and directions for future research

Our study carries some limitations. First, all measures utilized in the study originated from self-reports, including abusive supervision, without independent verification of supervisor behaviours. Future studies should consider incorporating diverse assessment sources, such as supervisor evaluations, peer ratings, or behavioural observations, to enhance reliability and validity and reduce potential bias. Second, data collection partially overlapped with the COVID-19 pandemic, a time when youth mental health challenges and suicide risk increased [54]. This temporal context is crucial in interpreting the findings and considering the potential impact of pandemic-related stressors on the participants’ mental well-being. A third limitation is that the study examined the mediating effects of thwarted belongingness and perceived burdensomeness separately to delineate their unique contributions. Future research could employ alternative methodologies to simultaneously investigate how these needs interact in influencing suicide risk development. A fourth limitation stems from our ethical obligation to exclude high-risk participant. While ethically imperative, this exclusion may limit generalizability of findings across the full suicidality spectrum. Future research should establish clinical collaborations (with appropriate safeguards) to address this constraint.

### Conclusions

Using the parallel-process latent growth model, a co-development relationship was identified between abusive supervision perceived by graduate students and their suicidal ideation, considering both their initial level and growth rate. Furthermore, the trajectory of perceived burdensomeness served as mediator in the impact of abusive supervision on suicidal ideation, as demonstrated by the latent growth curve mediation model. It underscores the significance of supervisor-student relationships and interpersonal psychological needs in crisis prevention among graduate students.

## Data Availability

Data supporting the results of this study are available from Y.Y., the corresponding author. The data cannot be made public because it contains information that could compromise the privacy of study participants.
